# Dysphagia is closely related to frailty in mild-to-moderate Alzheimer’s disease

**DOI:** 10.1186/s12877-023-04020-y

**Published:** 2023-05-17

**Authors:** Merve Güner, Arzu Okyar Baş, Serdar Ceylan, Zeynep Kahyaoğlu, Süheyla Çöteli, Pelin Ünsal, Çağatay Çavuşoğlu, Cemile Özsürekci, Burcu Balam Doğu, Mustafa Cankurtaran, Meltem Gülhan Halil

**Affiliations:** Faculty of Medicine, Department of Internal Medicine, Division of Geriatrics, Hacettepe University, Ankara, 06280 Turkey

**Keywords:** Dysphagia, Frailty, Dementia, Quality of life

## Abstract

**Introduction:**

Physical phenotype and the cumulative deficit model are two well-known concepts of frailty. One of the main components of frailty is loss of muscle mass and function, which may also include swallowing muscles, therefore is a risk factor for dysphagia. Since dysphagia is seen starting from the early stages of Alzheimer’s Disease (AD), in this study we aimed to reveal the relationship between frailty and dysphagia and dysphagia-related quality of life through Swallow Quality of Life (SwalQoL) tool in patients with AD and compare them with cognitively intact older adults.

**Methods:**

Comprehensive geriatric assessment, dysphagia evaluation by Eating-Assessment Tool (EAT-10) and SwalQoL questionnaire, and frailty assessment via FRAIL and Clinical Frailty Scale (CFS) were performed on all 101 participants of the study. Thirty-five patients were cognitively intact, 36 patients were diagnosed with mild AD, and 30 patients were diagnosed with moderate AD.

**Results:**

Sex distribution was similar between the groups, however, there was a statistically significant age difference. The prevalence of frailty increased according to both frailty indexes as the cognitive status deteriorated. All parameters of SwalQoL except fear and sleep parameters deteriorated as cognitive status impaired. In quantile regression of the total score of the SwalQoL questionnaire and multivariable logistic regression of EAT-10, frailty, as defined by CFS and FRAIL, was associated with dysphagia and poor quality of life regardless of age, presence of dementia, as well as nutritional status.

**Conclusion:**

Swallowing difficulties in AD negatively affects the quality of life, and it is closely related to frailty in mild-to-moderate AD.

## Introduction

Dysphagia which is the term to define difficulty in swallowing is a frequent problem in older age and becomes more prevalent in patients with dementia [1, 2]. Dysphagia may lead to serious complications including malnutrition, sarcopenia, and infections [3–5], and is related to decreased quality of life and increased risk of mortality [6]. Therefore, The European Society for Swallowing Disorders-European Union Geriatric Medicine suggests oropharyngeal dysphagia be accepted as a geriatric syndrome [7]. Dysphagia can be screened via Eating Assessment Tool- 10 (EAT-10) or the Swallow Quality of Life (SwalQoL) questionnaire [8, 9]. Dysphagia is a common problem in patients with Alzheimer’s Disease (AD). Even though dysphagia is expected to be more prevalent in moderate and severe AD [2], it is shown that swallowing problems and aspiration start from the early stages of AD [10].

Frailty is another common geriatric syndrome defined as a clinical state of increased vulnerability to intrinsic and extrinsic stressors and is associated with adverse outcomes like disability, hospitalization, and mortality [11, 12]. Low muscle strength, slow motor performance, exhaustion, low physical activity, and unintentional weight loss constitute the physical phenotype of frailty [13]. Another concept of frailty is the cumulative deficit model which is based on a comprehensive geriatric assessment (CGA) by counting the number of deficits accumulated, including diseases, physical and cognitive impairments, psychosocial risk factors, and common geriatric syndromes [14]. One of the main components of frailty is loss of muscle mass and function, which may also include swallowing muscles [15] Therefore, frailty can be accepted as a risk factor for dysphagia.

There is increasing evidence in terms of frailty-related dysphagia in recent years, it was stated that there is a strong association between deteriorated swallowing function and frailty in older adults [16]. This study aims to reveal the relationship between frailty and dysphagia, and its effects on quality of life in mild-to-moderate AD patients and to compare them with cognitively intact older adults.

## Material and method

### Study population

The study was conducted between 01 and 2020 and 01 May 2021 as a cross-sectional study. According to the power analysis [margin of error: 0.05 and confidence interval (CI): 0.80], the study group was determined as 65. After excluding patients who had one or more of the exclusion criteria, 66 patients aged 65 years or older with probable AD according to the National Institute of Neurological and Communicative Disease and Stroke/Alzheimer’s Disease and Related Disorders Association (NINCDS-ADRDA) criteria [17] admitted to the outpatient clinic of geriatrics were enrolled. Thirty-five cognitively intact patients were included in the study as the control group. CGA was performed on all patients. In addition, demographic data including age, sex, comorbidities, geriatric syndromes (such as osteoporosis, dementia, depression, urinary incontinence), medications, and history of falls were recorded for each patient. AD was staged with respect to Clinical Dementia Rating Scale (CDR) [18]. CDR score 0 was defined as normal cognitive status, 1 was categorized as mild AD, and 2 was classified as moderate AD [19].

### Exclusion criteria

The patients with the below conditions were excluded from the study;

- mild cognitive impairment (MCI),
- severe dementia defined with a CDR score of 3.
- other types of dementia including mixed types of dementia, vascular dementia, Lewy body dementia, frontotemporal dementia,
- other neurodegenerative diseases (Parkinson’s Disease, multiple sclerosis, stroke etc.)
- neuromuscular diseases,
- delirium and other psychotic diseases.

### Comprehensive geriatric assessment

Basic activities of daily living (ADL) (0–6 Points) [20] and instrumental activities of daily living (IADL) (0–8 points) [21] were recorded. Basic ADL includes bathing, dressing, transfer, toilet, continence, and feeding. Using the telephone, preparing food, housekeeping, laundry, transportation, using medications, handling finance, and shopping are evaluated for IADL. Mini-Nutritional Assessment Short Form (MNA-SF) (0–14 points) was used to assess the nutritional status [22]. The risk of malnutrition was defined as MNA-SF scores equal or lower than 11 points. Cognitive function and the presence of depressive symptoms were evaluated by Standardized Mini-Mental State Examination (SMMSE) [23], and Yesavage Geriatric Depression Scale (GDS) [24, 25], respectively. Scores ≥ 5 were accepted as depression risk according to GDS.

### Frailty assessment

The frailty scores of the patients were calculated via the *Fatigue, Resistance, Ambulation, Illness, Loss of Weight* (FRAIL) scale (0–5 points) [26], and *Clinical Frailty Scale* (CFS) (1–9 points) [27]. The FRAIL scale consists of 5 simple questions, presence of fatigue, muscle resistance, aerobic capacity, disease burden, and weight loss. Patients with ≥ 1 point FRAIL were defined as living with physical frailty. CFS is a semi-quantitative cumulative deficit tool that provides a generally accepted clinical definition of frailty. According to the clinical opinion of the physician, the scale that defines clinical frailty by giving a score between 1 and 9 (1: very fit; 2: well; 3: managing well; 4: living with very mild frailty; 5: living with mild frailty; 6: living with moderate frailty; 7: living with severe frailty; 8: living with very severe frailty; and 9: terminally ill). On the other hand, patients who scored > 3 points were accepted as living with frailty according to CFS [28]. Gait speed ≤ 0.8 m/s during a 4-m walking test using a manual stopwatch was used for determining the low physical performance [29]. Gait speed was calculated as the average of two measurements by the same physician.

### Swallowing assessment

Swallowing difficulty and its impact on quality of life were assessed by SwalQoL [8]. SwalQoL has 44 items in 11 domains as burden, eating duration, eating desire, frequency of symptoms, food selection, communication, fear, mental health, social functioning, sleep, and fatigue. Every item scored between 0 (the worst) and 100 (the best), The possible responses are “always” (0 points), “many times” (25 points), “sometimes” (50 points), “seldom” (75 points), and “never” (100 points). The score for each domain is calculated by adding the points of the responses to the questions in the domain and dividing the total by the number of questions in this domain. The total score has been calculated as the sum of the points of all domains and then divided into 11. We used the validated Turkish version of the SwalQoL [30]. The questionnaire was completed by the patients themselves or their caregivers. The other dysphagia screening tool, EAT-10 questionnaire which is a self-reported dysphagia questionnaire, was also performed on all participants [9]. A score ≥ 3 was accepted as the presence of dysphagia symptoms.

### Statistical analysis

Statistical analysis was executed by SPSS version 26.0. The categorical variables were presented as numbers and percentages. Normality tests were applied for continuous variables. Variables were presented as mean ± standard deviation (SD) or median [interquartile range (IQR)] concerning normal distributions. For comparisons of the data, the chi-square test or Fisher exact test was performed for categorical variables, with Bonferroni correction. Kruskal-Wallis test was wielded for continuous variables, where appropriate. Quantile regression analysis was used to assess the relationship between the total score of SwalQoL and age, sex, malnutrition, cognitive status, and frailty in different quantiles of 25th, 50th (median), and 75th percentiles. Two different regression models were formed to evaluate the possible factors that affect the total SwalQoL scores. The total score of the questionnaire was accepted as the dependent variable, coefficient and 95% CI were presented. n model 1 age, sex, presence of dementia, malnutrition, and CFS score were included. Model 2 consisted of age, sex, presence of dementia, malnutrition, and FRAIL Score. Multivariable binary logistic regression analysis (enter method) was wielded to evaluate the relationship between the presence of dysphagia symptoms screened by EAT-10 and age, sex, malnutrition, cognitive status, and frailty. Two different models were formed, in model 1 age, sex, the presence of dementia, nutritional status, and CFS score were included. Model 2 consisted of age, sex, presence of dementia, nutritional status, and FRAIL Score. Odds ratio (OR) and 95% CI were presented. In all analyses, p value < 0.05 was considered statistically significant.

## Results

One hundred one (101) patients were included in the study, and they were categorized as normal cognitive function (35 patients), mild AD (36 patients), and moderate AD (30 patients), according to their CDR scores. Sex distribution was similar between the groups (p > 0.05). The mean ± SD age of the cognitively intact group was 73.88 ± 6.76 years, and it was 75.58 ± 5.53 years in patients with mild AD. In patients with moderate AD, the mean ± SD age was 80.60 ± 7.99 years, and these age differences were statistically significant (p < 0.001). No difference was observed regarding chronic conditions except hypothyroidism (p < 0.001 for hypothyroidism, and p > 0.05 for the other chronic conditions).

The prevalence of frailty increased as the cognitive status deteriorated regarding both frailty indexes. Patients living with frailty were 14.3% and 32.4% in the cognitively intact group by CFS and FRAIL scales, respectively. The rate of patients living with frailty in mild AD was 41.7% by CFS and 44.1% by FRAIL; and 93.3% and 86.7% of patients with moderate AD were living with frailty according to CFS and FRAIL, respectively. These differences were statistically significant (p < 0.001 for both CFS and FRAIL). Geriatric syndromes including falls, incontinence, and malnutrition were more common in mild and moderate AD (p = 0.008 for falls, p = 0.001 for malnutrition and p < 0.001 for incontinence). ADL and IADL were mostly affected in moderate AD (p < 0.001 for both ADL and IADL) (Table 1).

**Table 1:** Demographic Features of Study Population and SwalQoL Scores According to Cognitive Status

		Cognitively Intact n = 35	Mild AD n = 36	Moderate AD n = 30	P
	**Age, years**	73.88 ± 6.76	75.58 ± 5.53	80.60 ± 7.99	< 0.001
	**Sex, Female**	18 (51.4)	23(63.9)	18.0(60.0)	0.56
	**Education, ≤ 5 years**	17(50.0)^a^	27(75.0)	26(86.7)	0.004
**Multimorbidities**	**Diabetes Mellitus**	18(51.4)	13(36.1)	7(23.3)	0.064
	**Hypertension**	27(77.1)	26(72.2)	22(73.3)	0.88
	**Hyperlipidemia**	12(34.3)	10(27.8)	7(23.3)	0.62
	**Coronary Artery Disease**	11 (31.4)	11(30.6)	10(33.3)	0.97
	**Hypothyroidism**	13(37.1)^a^	2(5.6)	2(5.6)	< 0.001
	**Malignancy**	6(17.1)	7(19.4)	2(6.7)	0.31
**Comprehensive Geriatric Assessment**	**Patients Living with Frailty,CFS**	5(14.3)^a^	15(41.7)	28(93.3)	< 0.001
	**Patients Living with Frailty,FRAIL**	11(32.4)	15(44.1)	26(86.7)^a^	< 0.001
	**Basic ADL**	6.0[1.0]	6.0[1.0]	3.0[3.0]	< 0.001
	**IADL**	8.0[0.0]	6.0[3.0]	1.0[2.0]	< 0.001
	**SMMSE**	28[5.25]	24[8.0]	17.0[9.0]	< 0.001
	**Yesavage GDS**	3.0[7.0]	3.0[3.0]	5.0[5.0]	0.016
	**Depression Risk (GDS ≥ 5)**	13(37.1)	9(25.0)	6(20.0)	0.28
	**Polypharmacy**	24(68.6)	28(77.8)	23(76.7)	0.63
	**Malnutrition** **Risk of Malnutrition**	3(8.6) 10(28.6) ^a^	6(18.2) 19(57.6)	8(27.6) 16(55.2)	0.001
	**Incontinence**	9(25.7) ^a^	19(57.8)	23(76.7)	< 0.001
	**Falls**	3(8.6)^a^	12(35.3)	12(40.0)	0.008
	Low Gait Speed (≤ 0.8 m/sec)*	21(60.0)	25(69.4)	27(90.0)^a^	0.024
	EAT-10(≥ 3)*	14(40.0)	23(63.9)^a^	27(90)^a^	< 0.001
**SWALQoL QUESTIONNAIRE**	**Burden**	100.0[0.0]	100.0[12.5]	87.50[28.13]	0.005
	**Duration**	100.0[12.5]	100.0[34.8]	75.0[56.25]	0.003
	**Desire**	100.0[16.7]	100.0[31.22]	79.15[35.38]	0.001
	**Frequency**	98.2[3.6]	94.60[12.03]	84.80[17.88]	< 0.001
	**Food Selection**	100.0[25.0]	100.0[37.5]	62.50[65.63]	< 0.001
	**Communication**	100.0[0.0]	100.0[0.0]	100.0[25.0]	0.029
	**Fear**	100.0[0.0]	100.0[4.69]	100.0[29.69]	0.20
	**Mental Health**	100.0[0.0]	100.0[3.75]	100.0[16.25]	0.015
	**Social**	100.0[0.0]	100.0[11.25]	90.0[28.75]	< 0.001
	**Fatigue**	75.0[50.0]	50.0[47.90]	41.65[50.0]	0.008
	**Sleep**	50.0[75.0]	64.5[75.0]	68.75[65.63]	0.94
	**Total Score**	88.73[12.28]	84.79[17.61]	77.72[15.72]	< 0.001

Variables are presented as n (%), Median[IQR] or mean ± SD

*CFS: Clinical frailty scale, FRAIL: Fatigue, Resistance, Ambulation, Illness, Loss of weight, ADL: activities of daily living, IADL: instrumental activities of daily living, SMMSE: Standardized Mini-Mental State Examination, GDS: geriatric depression scale, EAT-10: eating-assessment tool-10*

^*a*^ *According to subgroup analysis and after Bonferroni correction, the group of the difference was aroused from*

The patients were screened for dysphagia via EAT-10 questionnaire. In the cognitively intact group, 40% of the patients had positive screening results for dysphagia whereas the ratio of these patients was 63.9% and 90.0% in mild AD and moderate AD, respectively (p < 0.001). The total score of SwalQoL was also decreased as the cognitive status of patients got worsened (the median [IQR] total scores of SwalQoL were 88.73 [12.28], 84.79 [17.61], and 77.72 [15.72] in cognitively intact, mild AD and moderate AD, respectively). All parameters of SwalQoL except fear and sleep parameters deteriorated as cognitive status impaired. The most prominent differences were observed in symptom frequency (p < 0.001), food selection (p < 0.001), and social parameters (p < 0.001). Detailed results were shown in Table 1.

To investigate the factors that could affect dysphagia defined by EAT-10, a multivariable binary logistic regression analysis was performed. Frailty according to CFS increased the risk of dysphagia regardless of the age, sex, presence of dementia, and nutritional status in Model 1 (OR: 2.129, 95% CI:1.313–3.452 and p = 0.002). According to Model 2, frailty defined by FRAIL was associated with dysphagia (OR:1.567, 95% CI:1.075–2.884 and p = 0.019) (Table 2). In models 3 and 4, the presence of dementia was replaced with the stage of dementia, and CFS and FRAIL were independently associated with dysphagia (p = 0.011 and p = 0.046, respectively).

**Table 2:** Multivariable binary logistic regression analysis factors affecting dysphagia screened by EAT-10 (enter method)

Model		OR	95% CI		P-value
**1**	**CFS**	2.129	1.313	3.452	0.002
**2**	**FRAIL**	1.567	1.075	2.284	0.019
**3**	**CFS**	1.940	1.166	3.226	0.011
**4**	**FRAIL**	1.475	1.007	2.161	0.046

*OR: Odds ratio, CFS: Clinical frailty scale, FRAIL: Fatigue, Resistance, Ambulation, Illness, Loss of weight*

*Independent variables for Model 1 were Age, Sex, Presence of Dementia, Malnutrition, and CFS score, for Model 2 Age, Sex, Dementia, malnutrition, FRAIL Score; for Model 3 Age, Sex, Stage of Dementia, Malnutrition and CFS score; for Model 4 3 Age, Sex, Stage of Dementia, Malnutrition and FRAIL score*

For the parameters that may affect the overall score of SwalQoL, quantile regression models were developed. In the 25th, 50th, and 75th quantiles, CFS was negatively related to the total SwalQoL score for Model 1 (Coeff: -4.727, 95% CI: -7.799 to -1.655, p = 0.003; Coeff: -4.503, 95% CI: -7.063 to -1.943 p = 0.001; and Coeff: -3.037 95% CI: -3.037 to -0.177 and p = 0.028, for each quantile respectively). In model 2, there was a statistically negative and significant relationship between FRAIL scores and total scores in the 25th, 50th quantiles and 75th quantiles (Coeff: -3.472 95% CI:-5.743 to -1.201, p = 0.003, Coeff:- 2.593 95% CI: -4.535 to -0.650, p = 0.008 and Coeff:-1.534, 95 CI%: -2.343 to -0.726, p < 0.001, respectively). In models 3 and 4, the presence of dementia was replaced with the stage of dementia. In models 3 and 4, the presence of dementia was replaced with the stage of dementia, and CFS and FRAIL were independently associated with dysphagia and dysphagia-related quality of life (p = 0.013 at 25th percentile, p = 0.013 at 50th percentile and p = 0.038 at 75th percentile for CFS; and p = 0.021 at 25th percentile, p = 0.006 at 50th percentile and p = 0.005 at 75th percentile for FRAIL, respectively). In Table 3, regression analysis results were shown in detail.

**Table 3:** Quantile regression model; regression parameters estimating different quantiles (0.25, 0.50, and 0.75) of the total score of SwalQoL

Model		P25				P50				P75			
		Coeff.	95% CI		*P*	Coeff.	95% CI		*P*	Coeff.	95% CI		*P*
**1**	**CFS**	-4.727	-7.799	-1.655	0.003	-4.503	-7.063	-1.943	0.001	-1.607	-3.037	-0.177	0.028
**2**	**FRAIL**	-3.472	-5.743	-1.201	0.003	-2.593	-4.535	-0.650	0.008	-1.534	-2.343	-0.726	< 0.001
**3**	**CFS**	-4.210	-7.501	-0.919	0.013	-3.622	-6.467	-0.776	0.013	-1.584	-3.080	-0.088	0.038
**4**	**FRAIL**	-2.763	-5.102	-0.423	0.021	-2.621	-4.470	-0.773	0.006	-1.517	-2.533	-0.482	0.005

*Coeff: Coefficient, CI: confidence interval, CFS: Clinical frailty scale, FRAIL: Fatigue, Resistance, Ambulation, Illness, Loss of weight*

*The dependent variable for quantile regression models was the Total Score of SwalQoL.*

*Independent variables for Model 1 were Age, Sex, Presence of Dementia, Malnutrition, and CFS score, for Model 2 Age, Sex, Presence of Dementia, Malnutrition, and FRAIL Score, for Model 3 Age, Sex, Stage of Dementia, Malnutrition, and CFS score; for Model 4 3 Age, Sex, Stage of Dementia, Malnutrition, and FRAIL score*

## Discussion

According to our study, swallowing problems cause lower quality of life in mild-to-moderate AD patients than in cognitively intact patients. We found that dysphagia screened by EAT-10 and SwalQoL was associated with frailty independent of age, sex, presence of dementia, stage of dementia, and nutritional status.

People with dementia are susceptible to swallowing problems as a result of various health conditions. As they have a decline in their cognitive functions, they also have a loss in their body control. People affected with AD unrecognize the food, as a result of oral, visual, and tactile agnosia, and could not nourish themselves due to swallowing and feeding apraxia. Delayed oral transit time and pharyngeal response duration are other factors that cause deglutition problems. Therefore, during the disease process, patients may start to have difficulty swallowing leading to a progressive reduction in eating [2]. Decreased oral intake in dysphagia patients could lead to weight loss, dehydration, malnutrition, recurrent respiratory tract infections, and as a result decreased quality of life and increased mortality [31]. The prevalence of dysphagia in moderate to severe dementia is up to 93% [2], however, swallowing difficulties arise from the early stages of the AD [10]. Consistent with the literature, dysphagia was seen more frequently in our patients from the early stages in patients with AD.

In a systematic review published in 2016 by Madhavan et al., four risk factors for dysphagia (clinical disease history, age more than 70 years, depression, and physical frailty) were revealed [32], however, in that study, physical frailty was assessed by handgrip strength. In our study, it was revealed that physical frailty defined by the FRAIL scale was associated with dysphagia. Another study by Bahat et al. showed an association of dysphagia (screened by EAT-10) with frailty independent of age, sex, low handgrip strength, comorbidities, polypharmacy, and malnutrition [33]. Similarly, dysphagia screened via EAT-10 was related to malnutrition and frailty as a result of the study by Nishida et al. [34]. However, different from the previous studies in our study, dysphagia was also screened by SwalQoL, the frailty was assessed by both physical phenotype and cumulative indexes, and CFS was found to be mostly associated with dysphagia independent of age, sex, dementia, stage of dementia and nutritional status. Moreover, the quality of life of the patients was also evaluated in the present study, and it was stated that quality of life was affected by dysphagia mostly in patients with moderate AD.

In a study, frailty was defined as low functional status, increased comorbidity, low handgrip strength, living in a nursing home, and cognitive impairment which was related to oropharyngeal dysphagia in hospitalized patients with community-acquired pneumonia[35]. In another study ruled on community-dwelling adults, the non-ambulatory status of the patient predicted the aspiration[36]. On the other hand, the novel finding of our results was that cumulative frailty was associated with dysphagia independent of dementia and stage of dementia.

SwalQoL is a valid and reliable tool for screening oropharyngeal dysphagia since 2002 [8]. It’s superior to other screening tests because it evaluates the effect of swallowing difficulty on quality of life. The effect of dysphagia on quality of life was evaluated in many disorders that cause dysphagia like stroke [37], idiopathic Parkinson’s Disease [38], and other neurodegenerative disorders like multiple sclerosis [39]. However, to the best of our knowledge, our study is new and unique in this manner examining the impact of dysphagia on the quality of life in patients with AD. When comparing cognitively intact older persons, the disparities are apparent in the social, communication, and mental health subdomains of the questionnaire. It could be taken as dysphagia is more common and affects the quality of life in AD.

Our study has some strengths and limitations. Although the sample size is relatively small, nevertheless, a special patient group, patients with AD, was included, and also power analysis was conducted for the estimation of the study sample. Even though dysphagia and dysphagia-related decreased quality of life were independently associated with frailty, we could not comment on the cause-effect relationship between frailty and dysphagia because of the cross-sectional design of the study. Another limitation is that dysphagia was investigated only by the EAT-10 and SwalQoL. Since no instrumental methods for the certain diagnosis of dysphagia were applied, self-reported results are needed to confirm with the gold standard tests in large population prospective studies. Sarcopenia and other sarcopenia related parameters were also not assessed in this study, since sarcopenic dysphagia is another research area and we would like to emphasize the frailty and dysphagia relationship in patients with AD. However, our study has also some strengths. This is the first study to the authors’ knowledge, evaluating the quality of life related to dysphagia of the patients with AD screened by SwalQoL. All the patients in the study performed CGA and frailty assessment, and both physical and cumulative deficits frailty and dysphagia were found to be closely related regardless of the age, sex, nutritional status, and presence of dementia. Although frailty is a well-known factor in the emergence of dysphagia, our study is the first study to highlight the importance of frailty in the onset of dysphagia in patients with mild-to-moderate AD. Since the aforementioned limitations, our results could not be generalized for all patients with dysphagia.

In conclusion, we showed that older adults with mild-to-moderate AD have significantly lower scores of the SwalQoL for all domains than normal controls. Swallowing difficulties in AD negatively affect their quality of life, especially in the domains of burden, eating duration and desire, symptom frequency, food selection, communication, mental health, and fatigue. Furthermore, irrespective of the patient’s age, sex, nutritional and cognitive status, and even the stage of dementia, frailty (both physical and cumulative) were found to be closely related to dysphagia. Therefore, the evaluation of dysphagia in AD patients should contain a frailty assessment. Although difficult to say the causal relationship between frailty and dysphagia as a result and design of this study, patients with AD living with frailty should be screened earlier for dysphagia. Further long-term studies with a larger study population are needed to confirm our results.

## Data Availability

The data that support the findings of this study are available on request from the corresponding author, [M.G.]. The data are not publicly available due to. their containing information that could compromise the privacy of research participants.
