# Correction: Dysphagia is closely related to frailty in mild-to-moderate Alzheimer’s disease

**DOI:** 10.1186/s12877-026-07712-3

**Published:** 2026-06-06

**Authors:** Merve Güner, Arzu Okyar Baş, Serdar Ceylan, Zeynep Kahyaoğlu, Süheyla Çöteli, Pelin Ünsal, Çağatay Çavuşoğlu, Cemile Özsürekci, Burcu Balam Doğu, Mustafa Cankurtaran, Meltem Gülhan Halil

**Affiliations:** Faculty of Medicine, Department of Internal Medicine, Division of Geriatrics, Hacettepe University, Ankara, 06280 Turkey

**Correction: BMC Geriatr 23**,** 304 (2023)**

10.1186/s12877-023-04020-y

In the published article in BMC Geriatrics entitled “Dysphagia is closely related to frailty in mild-to-moderate Alzheimer’s disease” the cognitive assessment tool was incorrectly referred to as the “Mini-Mental State Examination (MMSE)” in the Methods section and in the related parts of the manuscript where the cognitive test was mentioned.

The error occurred due to the inadvertent use of the commonly known name of the test. However, as the copyright for the Mini-Mental State Examination (MMSE) is owned by Psychological Assessment Resources (PAR), the terminology should be corrected to “Standardized Mini-Mental State Examination (SMMSE),” which reflects the test used in the study.

Therefore, the text “Mini-Mental State Examination (MMSE)” and the abbreviation “MMSE” should be replaced with “Standardized Mini-Mental State Examination (SMMSE)” and “SMMSE,” respectively, throughout the manuscript.

The MMSE is a copyrighted instrument owned by Dr. Marshal Folstein and his company MMLLC (© 1975). PAR has the exclusive publication and licensing rights for the instrument, and is the registered trademark owner of the MMSE.

This correction does not affect the study data, statistical analyses, results, interpretation, or conclusions. The overall findings and conclusions of the published study remain unchanged.

The original article [1] has been corrected.
